# Reproductive Biology and Pollination Ecology of *Berberis lycium* Royle: A Highly Valued Shrub of Immense Medicinal Significance

**DOI:** 10.3390/plants10091907

**Published:** 2021-09-14

**Authors:** Susheel Verma, Ishfaq Ahmad Wani, Sajid Khan, Supriya Sharma, Priyanka Kumari, Prashant Kaushik, Hamed A. El-Serehy

**Affiliations:** 1 Conservation and Molecular Biology Laboratory, Department of Botany, Baba Ghulam Shah Badshah University, Rajouri 185234, India; waniishfaq680@gmail.com (I.A.W.); sajidkhan717@gmail.com (S.K.); sup.sharma111@gmail.com (S.S.); pk838511@gmail.com (P.K.); 2 Independent Researcher, 46022 Valencia, Spain; prakau@doctor.upv.es; 3 Department of Zoology, College of Science, King Saud University, Riyadh 11451, Saudi Arabia; helserehy@ksu.edu.sa

**Keywords:** *Berberis lycium*, variants, North-Western Himalayas, mixed mating, entomophily, seed longevity, anther movement

## Abstract

Study of reproductive biology and pollination ecology helps in understanding the life history patterns of species. Such a study brings to light the bottlenecks, if any, on account of which the individuals of the species are not able to reproduce in nature and ultimately helps in planning appropriate conservation strategies for the species under threat. The present study was aimed at examining the morphological and reproductive variance in *Berberis lycium,* a threatened ecological specialist growing within shrubberies and open hillsides of the North-Western Himalayas in India. *B. lycium* displays three different variants. Flowering period ranges from February to September. Pollen viability as reported on fluorescein diacetate and acetocarmine treatments was highest for variant I, while maximum pollen output was obtained for variant III. Pollen pistil interaction is brought by the movement of anther towards stigma. Fluorescence microscopy of hand pollinated club shaped stigma shows that the germinating pollen form a ring over the receptive adaxial surface. Pollination syndrome is entomophily. Variant II attracts a significantly large number of pollinators from diverse insect families. Breeding experiments reflect that plants are self-compatible and cross fertile. Reproductive output (% fruit set) was highest for variant II followed by III and I, respectively. This investigation helped to understand the effect of different biotic and abiotic constraints on the phenology and reproductive biology of the plant. The information generated so will enable conservationists to design appropriate strategies for its long-term survival and sustenance in nature.

## 1. Introduction

The entire Himalayan region is a repository of high value medicinal plants and is therefore, considered as a global biodiversity hotspot. Out of 18,440 plant species found in the Himalayan region, 8298 species are medicinally important [1,2]. *Berberis lycium* is one among them. Commonly known as Indian barberry, it is an evergreen shrub belonging to family Berberidaceae. It is a sub-erect branched shrub growing to a height of 3–4 m. It is native to Nepal and is abundantly distributed in the Himalayas extending through West of Pakistan, North and Central India, Afghanistan and the entirety of Nepal [3]. Within India, it is frequently distributed in Jammu and Kashmir, Uttar Pradesh, Madhya Pradesh, Himachal Pradesh, Uttrakhand, Sikkim and Nilgiri hills of Tamil Nadu within altitudinal gradients of 850–3500 m.a.s.l. [3,4,5]. This plant is used in polyherbal formulation of organized medicine such as Ayurveda, Siddha and Unani [6]. Pharmacological investigation of the entire plant body parts has revealed the presence of different secondary metabolites (alkaloids, tannins, saponins, flavonoids etc.) which signifies its potential role in the formulation of antibacterial, antifungal, antidiabetic and cardio-protective drugs [7,8]. Active constituents include berberine [9,10], an isoquinoline alkaloid which is widely known for its activity against severe diarrhea [11], cholera [12], latent malaria and amoebiasis [12]. Owing to its wide spectrum of medicinal significance this plant is being exploited at an alarming rate. Besides, it is being eradicated from the Himalayan region to reclaim the hill slopes for agriculture or to extract a valuable drug berberidine from its roots and stem [13,14]. Decline in the population of this species urges the need for its timely conservation so as to prevent this plant from getting critically endangered or extinct [15].

To design an effective conservation strategy for any species of plants, information about its reproductive behavior and pollination ecology along with other threatening factors becomes imperative [16,17]. Scientific data generated in such studies help in designing conservation strategies on one hand and in exploring the mechanism by which it has become endangered on the other [18]. In this context, study of the reproductive biology of the plants, particularly endangered and overexploited assumes a paramount significance. Reproductive biology allows us to understand the different phenophases of a species and processes beginning from the production of gametes to the germination of the seeds and constrains that limit the multiplication of the species in nature [19,20]. Studies on these aspects of reproductive biology of threatened taxa have provided valuable information regarding the limitation in the process of sexual reproduction and have helped in planning effective conservation and management programs [20,21,22,23,24,25]. Mating and the breeding systems of plant species have found to be detrimental in designing the genetic structure of the populations. Pollination biology is an important parameter in the life history of flowering plants that determines the success of sexual reproduction [26,27,28]. Effective conservation and management programmes based on the result of such studies have been executed for a number of threatened species [29,30,31,32,33]. Such studies bring to light the bottlenecks, if any, on account of which the individuals of the species are not able to reproduce in nature [34,35]. It also determines the genetic makeup of the populations, the magnitude of variations and therefore, ultimately helps in planning appropriate conservation strategies for the species under different categories of threat [36,37].

*Berberis lycium* Royle is one of the heavily exploited and threatened medicinal plants of the Himalayan region. Every part of the plant is used for medicinal purposes [38]. The species has been declared endangered in the North-West Himalayan region by FRLHT as per IUCN criteria of 2000 (envis.frlht.org/frlhtenvis.nic.in). Non-availability of basic biological data regarding phenology, reproductive biology, and pollination ecology of *Berberis lycium* are a major concern and are detrimental to its recovery plans. To fill these gaps, the present study was conducted to explore and gather the information on *B. lycium* with respect to above mentioned parameters, to understand this plant species at its various phenophases and to elucidate potential deficiencies in its reproduction and pollination mechanisms. This study helped to understand the effect of different biotic and abiotic constraints on the phenology and reproductive biology of the plant, including some unique mechanisms displayed by the flower to facilitate pollination and to ensure reproduction. Such a study will not only help to achieve improvement in the existing status of this species but also assist in unravelling the critical events which occur during the life cycle of the species and in turn help with planning strategies for effective conservation and management. The information generated thereof will enable conservation biologists to design appropriate strategies for long-term survival and sustenance of *B. lycium*.

## 2. Results

### 2.1. Phenology, Floral Organization and Traits

**Figure 1:**
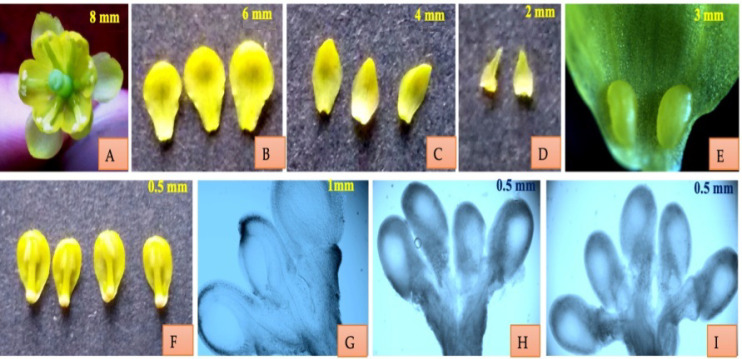
Floral structure of *B. lycium* (**A**) Hermaphrodite flower (**B**) Inner three larger sepals (**C**) Outer three smaller sepals (**D**) Two porophylls present over the calyx (**E**) Two nectaries present at the base of each petal (**F**) Anti-petalous, adnate and bithecous stamen (**G**–**I**) *Anatropous ovules* (three, four and five).

Flowers of *Berberis lycium* are bracteate, hypogynous, actinomorphic and hermaphrodite. Flowers grow in dense corymbose racemes. Flowers are bright, attractive and pale yellow in color, and grow in axillary clusters. Two porophylls are present over the calyx. Calyx in variants I and II is represented by 6 light yellow colored sepals in the two whorls of which the outer three are smaller than the inner three. In variant III calyx is represented by 2–3 whorls of sepals with number of sepals varying from 6 to 9. Corolla in variants I and II is bright yellow polypetalous having six petals. Corolla in variant III consists of 6–7 yellow colored petals. In flowers with 7 petals, as many as 7 stamens are present. Two nectaries are present at the base of each petal. Six antipetalous, adnate and bithecous stamens constitute the androecium in all the three variants. An anther has two lobes, each with two unequal small pollen sacs (SPSs) and large pollen sacs (LPSs). Gynoecium is represented by a single pistil which is differentiated into stigma, style and ovary. Stigma is round with a depression in the middle. It is wet and has a copious number of exudates. Style is hollow and short. Ovary is unilocular having three to five anatropous ovules. Number of ovules in variant III is usually 5 varying from 4 to 6. The fruits are berries, ovoid in shape and acquire purple or bright red color upon maturity. The size of berries may range from 8.2 ± 1.4 mm, 5.1 ± 1.1 mm to 6 ± 0.9 mm, 5.5 ± 1mm in length and diameter, respectively. Weight of the fruits range between 303 ± 24 and 209 ± 19 mg. Seeds are yellow to pinkish in color and on average the fruit contain 2–5 seeds (Figure 1). 

#### 2.1.1. Variant I

This is the predominant type observed. The plant bodies are differentiated into branched root and shoot system. Leaves arise alternately on the shoots in clusters. Their number varies from 3 to 9 per cluster. They are lanceolate with entire or spinous margins. Each node on the shoot bears one trifid spine. Inflorescences are racemose and arise from the axils of the leafy shoots in the last week of February. Peak flowering occurs in the middle of March. A bud takes about 20–22 days to transform into a flower after it appears on the inflorescence. Flowering completes by first week of April. 

#### 2.1.2. Variant II

These plants enter the reproductive phase in April and are marked by the presence of relatively long inflorescences. Leaves are papery and spathulate in shape with apical spine. Buds begin to open in the month of May in an acropetal succession in an inflorescence. Peak flowering was seen in the third week of May and completes by the second week of June. An inflorescence bears about 30 flowers on an average reaching up to 50 per inflorescence.

#### 2.1.3. Variant III

Plants enter the reproductive phase in the month of September and continue producing flower till January. The leaves are either entire or with spines only towards the apical end of the lamina. Leaves arise in an alternate manner. Number of leaves at each node varies from 3 to 10. Leaves are also present on the inflorescences. Inflorescences of different sizes were present on a single plant ranging from 3 to 10 cm. Leaves are also present on the inflorescence. Number of sepals and petals varies ranges from 6 to 9 and 6 to 7, respectively. Number of ovules is usually 5. Detailed floral and morphological differences in different variants of *B. lycium* are provided in Table 1. One-way ANOVA showed that there was a significant difference in the majority of the morphological characters in different variants and the level of significance ranged from < 0.001 to <0.05. However, a number of other traits, such as width of the leaf, width of the bract, length and width of the porophylls, size of petal, length of stamens and width of flower, were not statistically different. 

### 2.2. Anthesis and Pattern of Flowering in Inflorescence

Flower anthesis starts when the petals of the flower start to unroll and separate from each other. This activity was seen during separate time periods for different variants. Flowers begin to open between 06:00 and 08:00 am and continue to open throughout the day with maximum number of flowers opening between 09:00 am and 04:00 pm. Increase in the mean day temperature results in early flower anthesis. In variant III, flowers buds start to open at 06:17 am followed by variant II at 07:00 am and variant I at 08:00 am, respectively. Flowers open randomly, exhibit dichogamy and are protogynous. On an average, a flower takes about 3 h to open completely (Table 2). Flowers continue to open throughout the day. None of the flowers open during the night. Anther dehiscence starts when the flowers are about 0.4–0.5 cm across. In some flowers, anthers dehisce once the flower has fully opened. Anther lobes dehisce sequentially one by one on alternate stamens. After about three days of pollination, the flowers start withering and on the fourth day, the fertilized red color pistil is exposed. Analysis of variance (ANOVA) showed that different variants differed significantly (*p* < 0.05) with respect to flowering, time and maximum anthesis while the other characters were statistically non-significant.

### 2.3. Stigma Receptivity

**Figure 2:**
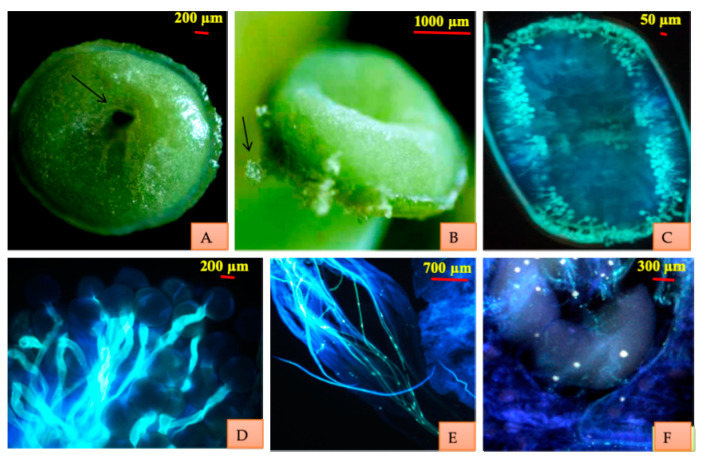
Stigmatic surface and receptivity. (**A**) Stigma showing the depression in the middle which merges to hollow style. (**B**) Pollen trapped on wet stigmatic surface. (**C**) Pattern of pollen germination on the stigma. (**D**) Pollen germination. (**E**) Pollen tubes traversing through style. (**F**) Pollen tubes entering the ovules.

Fluorescence microscopy of the manually pollinated stigma (at different time intervals under Nikon Eclipse 80 Fluorescence microscope) reflects that stigma becomes receptive in the bud condition about three days before anthesis. It remains receptive for 26–28 h prior to anthesis. Stigma is green, wet and receptive on the day of anthesis. It is round with the depression in the middle. While still green in color, stigma loses receptivity after 28h of opening. Stigma acquires brown color 6–7 days after anthesis and is persistent. Fluorescence microscopy of the pistil showed the germinating pollen on the stigma, pollen tubes moving through the stylar tissue and entering the ovule through the micropyle (Figure 2). The pollen tubes reached the ovary within 30–40 min. 

### 2.4. Pollen Output, Viability and Pollen Ovule Ratio

**Figure 3:**
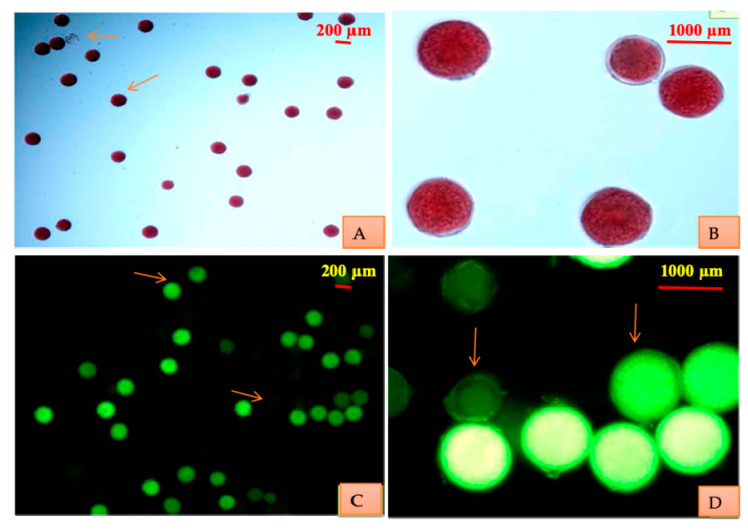
Pollen viability observed after treatment with (**A**,**B**) 1% acetocarmine (**C**,**D**) 1% fluorescein diacetate (FDA).

Pollen grains are spherical with smooth exine. Pollen output per anther and per flower is 1329 (±317.30) and 7974, respectively, for variant I. For variant II, pollen output for a single anther and the flower is 1002 ± 244.84 and 6012, respectively. Pollen viability as tested with 1% acetocarmine and flourescein diacetate (FDA) varied from 74.60% to 94.96% and 75.68% to 92.76%. Pollen ovule ratio stands at 1002:1. In variant III, single anther produces about 1434 ± 198.98 pollens. A flower with 6 and 7 stamens produces about 8604 and 10,038 pollens, respectively. Pollen ovule ratio is 1720:1 (Table 3 and Figure 3). One-way ANOVA showed that both pollen viability and pollen output per flower show significant variation among different variants and were significant at *p* <0.001 level (Table 3)

### 2.5. Mechanism of Anther Dehiscence

**Figure 4:**
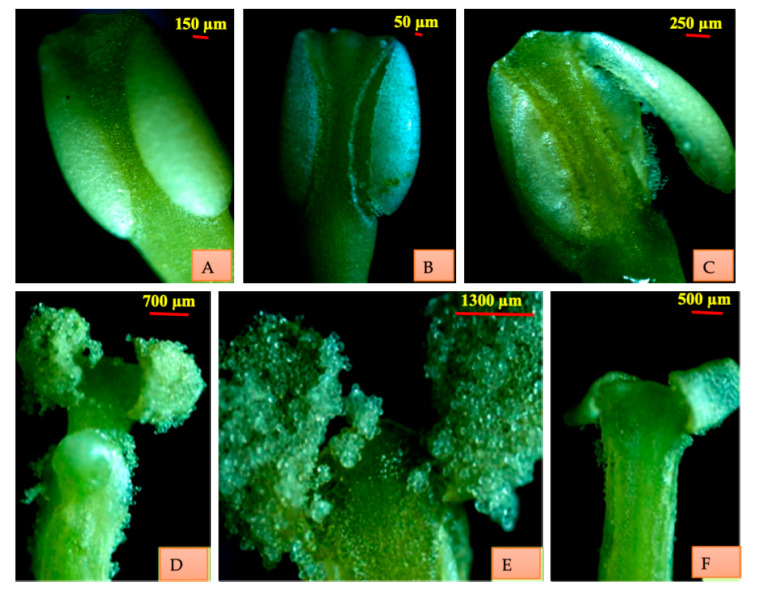
Mechanism of anther dehiscence. (**A**) Intact anther lobes. (**B**) Separation of anther along the line of dehiscence. (**C**,**D**) Outward movement of anther lobes. (**E**) Wet and sticky pollen grains. (**F**) Empty anther.

In the bud condition, stamens are closely adpressed to the pistil. As the flower opens, stamens move away from the stigma. In the fully opened flowers, stamens are placed against the corolla lobes, one each. Anther dehiscence occurs simultaneously with the anthesis. As the flower starts opening, anthers of the alternate stamens start dehiscing. Stamens with the fully dehisced anthers remain close to corolla lobes. Large pollen sac (LPS) of one lobe of the dehiscing anther starts moving away from the filament and becomes almost perpendicular to the long axis of the stamen. This is achieved in 10–15 min. It then turns inwards and goes upwards exposing the inner pollen laden surface of the stigma assuming an angle of 180° vis-a-vis axis. This is accomplished in 3–5 min. Ultimately, it curves towards the tip of connective. At this stage anther lobe is at the level but apart (3mm) from the stigma (Figure 4).

### 2.6. Pollination Ecology

**Figure 5:**
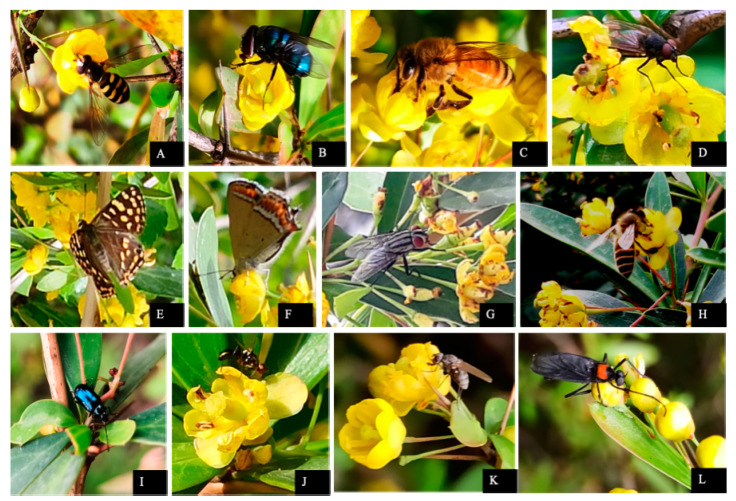
Insect visitors of *Berberis lycium*. (**A**) *Eupeodes luniger*; (**B**) *Calliphora vomitoria*; (**C**) *Apis mellifera*; (**D**) *Calliphora* sp.; (**E**) *Dodona durga*; (**F**) *Heliophorus sena*;(**G**) *Sarcopha* sp.; (**H**) *Apis cerana*; (**I**) *Altica* sp.; (**J**–**L**) *Plecia* spp. * Images captured with Nikon D7000 DSLR camera (Nikon, Tokyo, Japan).

Hanging slide experiments rule out the possibility of wind pollination (anemophily). *Berberis lycium* is insect pollinated (entomophily). Insect visitors belong to 4 orders (Hymenoptera, Diptera, Lepidoptera and Coleoptera), 14 families and 17 genera. Hymenoptera was observed as the most dominant order with nine pollinator species (Figure 5). *B. lycium* has all the contrivances (brighter color, fragrance, nectar and pollen) for entomophily. Pollen deposition on the stigmatic surface requires the intervention of insects. *Apis cerana* and *A. mellifera* are predominant pollinators.

When the proboscis of the nectar foraging insects touches the base of the stamen with the dehiscing anther, it pushes the stamen towards the stigma. This way the anther touches the stigmatic surface, thereby depositing a huge amount of pollen on the stigma. The anthers remain stuck at the stigma for 22–30 s and the stamen slowly retrieves back to its original position within 15 min. It was observed that the stamen with the dehiscing anther lobes gets pushed towards the stigma instantaneously thereby affecting the pollen deposition on the stigma. Pollinator abundance and richness were highest for variant I (Table 4). 

**Figure 6:**
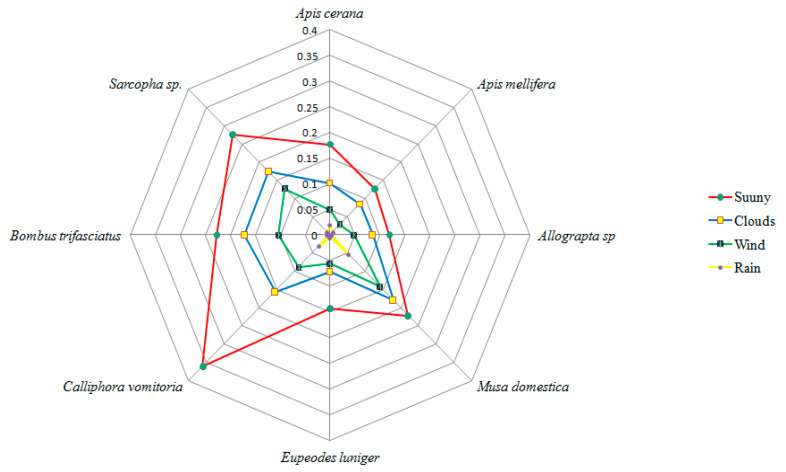
Effect of different environmental factors on insect visiting efficiency (measured following the methodology of Yaqoob and Nawchoo [19]).

On the basis of insect visiting efficiency indices, *Calliphora vomitoria*, *Bombus trifasciatus*, *Sarcopha* sp., *Apis cerana*, *Apis mellifera*, *Musa domestica*, *Apis mellifera*, *Eupeodes luinger*, seem to be the dominant pollinators while *Dodona durga*, *Heliophorus sena*, *Formica fusca*, *Coelixys* sp., *Praezygaena caschmirensis* were designated as infrequent visitors. Comparative indices of different insect visitors are provided in Table 5. On normal sunny days with greater illumination and suitable weather conditions, insect foraging starts early in the morning. Highest visitation of the insects was seen between 12:00 and 15:00h which declined thereafter. External environmental factors such as wind, clouds, rain and temperature showed a significant influence on the visiting behavior of insect pollinators. The highest insect visiting efficiency of pollinators was reported on bright sunny days, which declined because of clouds, wind and rain (Figure 6).

**Figure 7:**
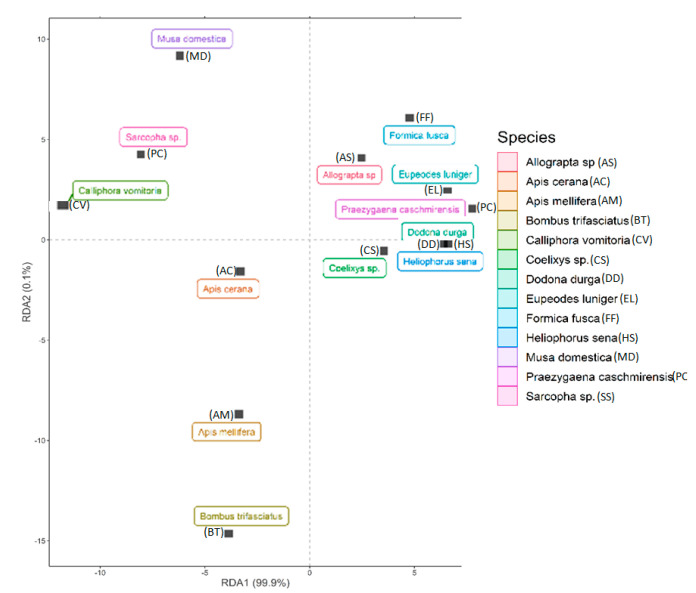
Redundancy analysis (RDA) plot of the studied pollinators based on different pollination indices.

**Figure 8:**
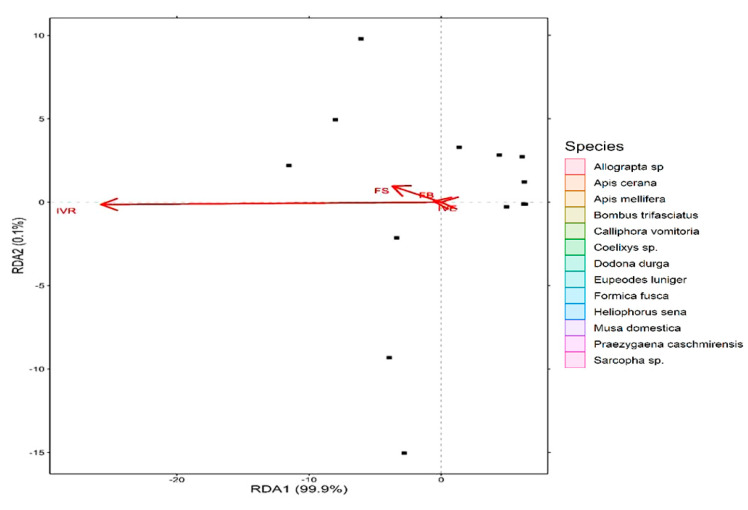
Redundancy analysis (RDA) biplot showing the relationship between studied plant pollinators and the pollination indices contributing greater than 40% variation in the data.

The redundancy analysis (RDA) based on different pollination indices classified different insect visitors into several groups. *Apis* sp., *Calliphora vomitoria, Musa domestica,* *Sarcopha* sp. consists of single isolated species while the rest of the pollinator species were clustered together into same group and show varying degrees of overlap. Further, the first axis (RDA 1) explained about 99.9% of variation in the dataset while the second axis (RDA 2) explained about 0.1% of the variation (Figure 7). Additionally, all the studied pollination indices revealed greater than 40% variation in the data with the highest being reported for index of visitation rate (r = 1), foraging speed (r = 0.99), foraging behavior (r = 0. 96) and insect visiting efficiency (r = 0.85) (Figure 8).

### 2.7. Breeding System and Reproductive Output

Flowers were studied for open, unassisted and manual pollinations. In case of the open pollination, flowers on 25 inflorescences on 15 plants were tagged and observed for pollen load and pollen germination on the stigma of flowers. Pollen load in open pollinated flowers varies from 68 to 884. Percentage pollen germination varied from 93% to 100%. Different experiments on breeding behavior reveal that *B. lycium* is self-compatible and cross fertile. None of the emasculated and bagged flowers set fruit, indicating that no apomixis or agamospermy occurs in *B. lycium*. Manual self-pollination confirms autogamy as well as geitonogamy. The highest values of percent fruit set were seen for variant II. The highest number of percentage fruit set was observed for manual cross pollination treatments (VIIMCP: FR = 100, %FS = 71, *p* < 0.001) followed by manual geitonogamy (VIIMG: FR = 100, %FS = 61 *p* < 0.001). Lowest values for percent fruit set in variant II were calculated for open pollination (VIIOP: FR = 100, %FS = 52 *p* < 0.001). Detailed results of the breeding system are explained in Table 6. 

The results of the two-way ANOVA (analysis of variance) indicate that there is a significant effect of different treatments (natural and manual treatments) on both the fruit and seed development, but the effect of different variants turned out to be non-significant (Table 7).

**Figure 9:**
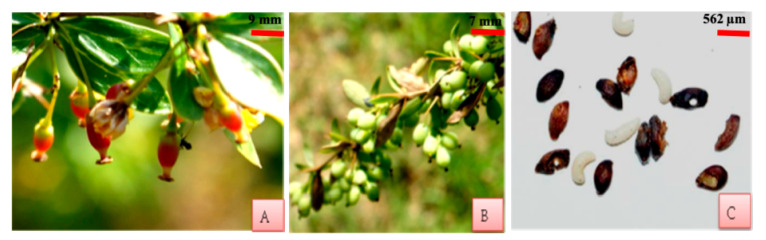
(**A**,**B**) Fruit formation in *Berberis lycium*; (**C**) damage caused to seeds by larvae of the insects.

The fruits start ripening in the last week of May and continues to ripen until the middle of June. Fruits are called berries and are globose or sub-globose in shape. The fruits are initially red in color, then turn green and become purplish on ripening. Percentage fruit set is higher in manual cross pollination (MCP). A large amount of fruit gets aborted. Two kinds of fruit appeared on the inflorescences, one without seeds and other with 1–5 seeds. The fruits without seeds turn black while the fruits with seeds invariably get damaged by some infection. The seeds of such fruits get exposed before they mature. Flowers subjected to unassisted pollination did not bear any fruit. Some flowers get infested by insects whose larvae voraciously feed on pollen, thereby limiting the self-pollination to the stigma. It was observed that such flowers did not bear any fruit. Percentage fruit set and seed set per plant in nature was 66.66% and 58.33%, respectively. However, nearly 10% of the seeds were nonviable due to damage caused by larvae of an insect (Figure 9).

### 2.8. Seed Germination and Longevity

**Figure 10:**
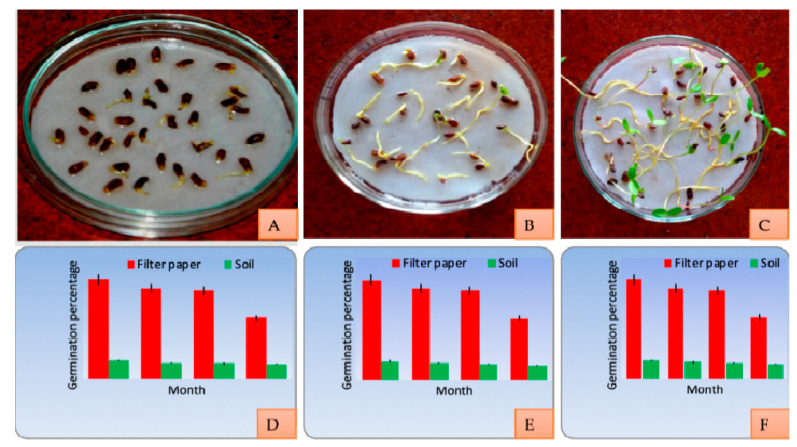
(**A**–**C**) Seed germination in *Berberis lycium*; (**D**) seed longevity in variant I; (**E**) variant II; (**F**) variant III.

Seeds were kept for germination in the months of August, September, October and November. Highest germination percentage was reported for seeds on filter paper as compared to seeds sown within the soil. Seeds kept on the wet filter paper started germinating after 4 days and by the 8th day all the seeds germinated while the seeds within the soil started germinating from the 9th day. These seeds took about 15 days to germinate in soil. The ANOVA (analysis of variance) depicts that the germination potential of the seeds does not show a significant variation within different variants. Seed longevity shows a drastic decrease (100% to 55% on filter paper and 20% to 14% within soil) with respect to increase in the storage period (Figure 10). 

## 3. Discussion

Occurrence of variants of a species in an area indicates the diversification of the species. All the three variants of *Berberis lycium* observed in the study area are characterized by different foliar characters. They differ with respect to size, shape and texture of leaves, inflorescence length, number of flowers per inflorescence, diameter of flowers and number of floral parts. Earlier, Ahrendt [39] recognized four variants of *Berberis lycium* of which three have been reported from Kashmir. The plants of variants I and II enter the flowering phase in the months of February and April, respectively, and the flowering period lasts for just one month whereas the plants of variant III enter the reproductive phase in September and continue producing flowers till January. Variation in flowering time is often considered with a genetic component and has been demonstrated in many studies by different workers [40,41,42,43,44]. 

For the successful initiation of pollen pistil interaction, pollen viability and stigma receptivity are important parameters [45,46,47,48]. Presence of certain enzymes such as acid phosphatases, peroxidases and esterases mark the receptivity of stigmas [49,50]. Extended stigma receptivity assures pollination, fertilization and reproductive success in plants [51]. The stigma remains receptive for greater periods in *B. lycium* to provide proper conditions for pollen to germinate and affect fertilization. This time frame appears to be an approach to ensure reproductive output in this plant species. Mechanism of anther dehiscence is unique and similar in all the three variants. Anther dehiscence occurs simultaneously with anthesis. The large pollen sacs of dehiscing lobes of alternate anthers detach themselves from the filament remaining attached at the top of the connective part and then move in such a way that pollen laden sacs face the stigmatic surface to affect pollination. This pattern of anther dehiscence is believed to be associated with the size difference between the dorsal and ventral side of pollen sacs in each theca, the ventral sacs being much smaller than the dorsal one. The stomium stretches longitudinally between the two pollen sacs and around the base and upward along the dorsal side of the theca which permits the wall of dorsal pollen sac to curve upward at dehiscence [52]. Alternate anther dehiscence appears to be an adaptation for efficient and extended pollen availability to the pistil.

Like animals, plants also respond to mechanical stimuli, although the signaling machinery is poorly understood [53]. Flowers and leaves are more commonly responsive to external mechanical stimuli among plant organs [53]. The first report of thigmo-response of stamens dates long back when Linnaeus described the sensitive stamens of *Berberis vulgaris* flowers in his 1755 version of the *Flora Suecica*. Stamen movement determines the fate of pollen transfer with pollinators [54]. Staminal movement is normally correlated with cross-pollination in plants [55] though it also promotes self-pollination [36] as has been observed in *B. lycium*. A characteristic feature of sensitive stamens is the presence of papillate structures in the sensitive areas which are believed to be related to the water mobility after being irritated and, thus, inducing the thigmonastic response [56]. In *B. lycium*, only the basal part of stamens is sensitive, papillae are localized only at the base of staminal filament.

The floral morphology of different plant species determines their pollination behavior [57,58]. Different floral traits such as fewer terminal leaf panicle inflorescences, small perianth, exposed stigmatic surface and raised anthers, delicate fragrance, nectar availability, and dense and showy inflorescences favor insect pollination [58,59]. Our observations propose that the pollination syndrome of *B. lycium* is entomophilous; i.e., it is pollinated by insects. In all the three variants, pollination is dependent on insect visit. When the visiting insect inserts its proboscis to suck nectar secreted by nectaries at the base of corolla lobes, it invariably touches the base of staminal filaments and induces their movement, thus pushing them towards the interior of flower in such a way that the dehisced anther lobes deposit pollen on the receptive stigmatic surface. The insects with strong proboscis can induce the staminal movement. Morphotype II attract diverse numbers of pollinators as compared to other two morphotypes which is attributed to greater length of inflorescences and large number of flowers. Floral secretions, colorful and attractive inflorescences are more imperative in attracting the pollinators than aroma [60,61]. The color of flowers could also be considered as an advertisement of reward when the meaning of flowers is to attract pollinators. Bright yellow petals of *B. lycium* have evolved as a means for attraction of pollinators. Pollinators prefer warmer afternoon hours to visit the flowers after which their visitation frequency decreased. Such findings draw their support from the reports of [42]. Different plant species receive a drastic decrease in the visitation efficiency of pollinators during wind, cloudy periods and rain [62,63]. Adverse climatic conditions were found to hamper the visitation frequency and efficiency of the pollinators.

The mechanisms of anther dehiscence, staminal movement and pollination syndrome appear to be an adaptation to affect the self-pollination in *B. lycium.* It is further strengthened by low fruit set in flowers whose self-pollen is foraged by predatory larvae. Results of pollination experiments in *B. lycium* point towards the mixed breeding strategy followed by the species to ensure reproductive output. This kind of strategy not only ensures sexual reproduction but also generates variation and distributes it effectively [64]. Low fruit set in variants I and III in open pollination can be due to non-availability of pollen grains to receptive stigmata. Both pollinator and pollen limitation appear to be the reasons for low reproductive output in open pollination in variants I and III. Seed set was high in variants I and II. However, the seeds are predated by larvae of some insects in both cases. Variant I is more susceptible to predation leading to 10% loss in reproductive output. Majority of fruits of variant III were seedless and those with seeds get infected and damaged. Seeds in such fruits thus turn nonviable leading to low reproductive output. Low reproductive output of this variant appears to be the reason for its low representation in the study area.

The percentage seed germination varied greatly on wet filter paper and in soil conditions. Seeds fail to germinate and emerge out of the soil when sown in soil. Low germination of seeds sown in soil can be attributed to non availability of light to the germinating seeds. Many workers have reported this in different weed species [65,66,67]. It has been reported that very little light is reached to the seeds below a depth of 4mm in all soil types [68]. Increase in the storage period and aging of seeds results in decreased vigor and germination index [69]. Decrease in the germination percentage with respect to their increased storage period reflects the decreased longevity of the seeds in *B. lycium*. These results are in agreement with the findings of Arif [70] who reported inverse relation between seed longevity and storage period. The differences in the germination percentage of seeds may be due to decline in phospholipids and polyunsaturated fatty acids [71]. Our results are equally supported by the findings of Kandil [71] and Muhammadi [72] who also reported significant decrease in the germination percentage of seeds with respect to increased storage periods.

## 4. Materials and Methods

### 4.1. Phenology, Floral Organization, Traits and Reproductive Biology

Mature and healthy plants in full bloom were randomly selected and tagged to study floral traits and biology. A detailed study regarding different quantitative characteristics of floral parts, viz., length and width of the flower, porophylls size, length of pistil, length of stamen, was carried out under a stereo zoom microscope (Nikon- BX51). The number of flowers per inflorescence, number of anthers per flower, length of inflorescence was noted directly in the field with applied standard deviations.

Data on different aspects of reproductive biology like plant and floral morphology, phenology, pollen viability, pollen–ovule ratio, anther dehiscence and pollination mechanism, pollinator behavior, pollen–pistil interaction, reproductive output and seed germination were collected for the plant under study.

### 4.2. Floral Biology

Data on flower opening (anthesis) were collected, both in nature and under lab conditions. Time and pattern of anther dehiscence was determined by periodic examination of anthers. Stigma receptivity was determined by manually pollinating the stigmata and examining them for pollen germination at different time intervals either directly or after fixing them in Carnoy’s fixative (three parts absolute alcohol and one part glacial acetic acid) and then shifting them to 70% alcohol. Pistils were then stained in Lewis’ stain (mixture of 2mL of aqueous acid fuschin, 2mL of 1% aq. Lightgreen, 40 mL of lactic acid and 46 mL distilled water) [38] for microscopic studies. Some of the pistils were stained with Aniline blue for fluorescence microscopy [73].

### 4.3. Pollen–Ovule Ratio

Pollen output was calculated by first counting the number of pollen grains per anther and multiplying this figure by the number of stamens per flower. The ovule count was determined by putting the ovaries in 4N NaOH for 12–14 h at 60 °C in a hot air oven, washing them thoroughly in water to remove every trace of NaOH and then gently squashing the ovaries in a drop of Lewis stain on a slide and then counting ovules by observing these under the microscope. The amount of pollen divided by number of ovules per flower gave pollen–ovule ratio. Pollen–ovule ratio was calculated following the method of Kumari [18].

$$P/O=\frac{Number\;of\;pollen\;grains\;per\;anther\times Number\;of\;anthers\;per\;flower}{Number\;of\;ovules\;per\;flower}$$

### 4.4. Pollen Stainability and Viability

Pollen stainability and viability was checked by conducting a stainability test in 1% acetocarmine and FCR (a mixture of FDA in acetone and sucrose) test, respectively. The concentration of sucrose used was 10%. In the stainability test, pollen with stained cytoplasm were considered viable while shriveled and unstained were taken as nonviable. In the FCR test, pollen with fluorescent and non-fluorescent cytoplasm were treated as viable and nonviable, respectively.

### 4.5. Pollen–Pistil Interaction

Pollen–pistil interaction was studied by following the Aniline blue fluorescence method [18]. Pistils were cleared by keeping them in 4N NaOH at 60 °C for 3 h. They were then stained in 0.005% aniline blue (discolor) for 30 min. Later on, they were mounted in 1:1 *v/v* mixture of aniline blue and glycerin and observed under microscope (Nikon 80 i Eclipse (Nikon, Tokyo, Japan)). Under fluorescence microscope, germination of pollen on the stigma and path of pollen tubes through the stylar tissue to the ovule was studied.

### 4.6. Pollination Mechanism

To understand the pollination mechanism, different structural features of the flower like type of corolla, position of the reproductive parts in a fully opened flower and kind of pollen and stigma were studied carefully. To check anemophily, slides smeared with Mayer’s albumin were suspended at varying distances from plants. These slides were then examined after 24 h by staining in Lewis stain for presence or absence of the pollen of the plant under study. To check entomophily as the possible mode of pollination, observations were made whether or not the flowers are visited by the insects. The rewards offered by the flowers to the visiting insects were also noted. Behavior of the insects visiting the flowers was monitored to determine how they affect pollination. 

Different pollination indices were calculated for all the pollinators to determine their contribution to pollen transfer. The foraging behavior (FB) of insect visitors was ascertained through direct field visitations at regular intervals of time. It was determined as the time spent by a particular pollinator per inflorescence per visit [19,74]. Foraging speed (FS) was calculated as the average number of flowers visited per minute of time [19,75]. Insect visitation efficiency (IVE) and insect visitation frequency (IVF) were determined following the methodology put forward by Yaqoob and Nawchoo [19].

### 4.7. Mating or Breeding System

Different pollination experiments were carried out to determine the nature of breeding system. Inflorescences were subjected to forced selfing by bagging them. Compatibility status was determined by manual pollinations (manual self and crosspollination). In any pollination experiment, fruit set was taken as the end product of effective pollination, thereby confirming the compatibility. In order to ascertain breeding behavior for *B. lycium* the following experiments were placed:

- Control
- Flowers were kept undisturbed and monitored for fruit set.
- Agamospermy or apomixis
- During bud condition flowers were emasculated and bagged to check fruit set.
- Xenogamy
- Flowers were emasculated, followed by manual cross pollination and bagging.
- Autonomous selfing or unassisted selfing
- Flowers were bagged during bud condition and monitored regularly for fruit set.
- Facilitated autogamy (selfing)
- Plants were emasculated, followed by manual self-pollination and bagging.
- Geitonogamy

Stigmas of the flowers were pollinated with pollen from different flowers of the same plant.

### 4.8. Reproductive Output

For calculating reproductive output, percent fruit set per plant and percent seed set per fruit were calculated. Both these aspects were studied for open pollination in nature. To determine percent fruit set, first the average number of flowers per plant and then average number of fruits formed per plant was determined. Percentage fruit set was calculated using the following formula:

$$Percentage\;fruit\;set\;=\;\frac{Average\;no.\;of\;fruits\;formed\;per\;plant}{Average\;no.\;of\;flowers\;produced\;per\;plant}\;\times \;100$$

For determining percentage seed set, first the average no. of ovules per ovary and then average number of seeds per fruit was calculated. Percentage seed set was determined using the following formula:

$$Percentage\;seed\;set\;=\;\frac{Average\;no.\;of\;seeds\;per\;fruit}{Average\;no.\;of\;ovules\;per\;ovary}\;\times \;100$$

### 4.9. Seed Germination

The potential of the seeds to germinate was determined by keeping the seeds for germination under lab conditions and in substrata like moist silver paper and mixture of sand, manure and garden soil in the ratio of 1:1:1. Total number of germinated seeds by total number of seeds kept for germination yielded the percentage seed germination.

### 4.10. Data Analysis Using Statistical Software

The data analysis was performed using R software v.4.0.3 (R Core Team, 2021). We performed one-way ANOVA (analysis of variance) test to study whether the studied variants differed in terms of their morphological characters, pollen biology and floral anthesis. Two-way ANOVA test was carried out to determine the simultaneous effect of different variants and treatment on fruit and seed development. Redundancy analysis (RDA) was carried out to study the similarity between pollinators in terms of their pollination indices separately using the *Biodiversity R* package. Additionally, we searched for the most contributing factors, which explained 40% or more of variation in the foraging behavior and pollen pollinator efficiency, respectively, and thus were most effective in separating different pollinators.

## 5. Conclusions

On the basis of the present study related to reproductive biology and pollination ecology of *Berberis lycium*, it is concluded that this plant species exhibits tremendous morphological variability as reflected through the occurrence of multiple variants. Although the plant is self-compatible, deposition of self-pollen on stigmatic surface requires the insect intervention wherein the visiting insect stimulates the movement of the stamens towards the stigma to facilitate pollination. The pollinators, therefore, play a dual role in the process of sexual reproduction by promoting self-pollination as well as acting as a carrier of pollen to facilitate cross pollination. Pollen and pollinator limitation can lead to low reproductive output or reproductive failure in the species. Greater reproductive output in variant II under open pollination is the outcome of diverse pollinator attraction and visitation to larger inflorescences. Further the decline in the reproductive output of the species is supplemented by infestation of predating insects.

## Figures and Tables

**Table 1:** Variability among three variants of *Berberis lycium* with respect to different floral traits.

S. No.	Parameter	Variant I	Variant II	Variant III	Mean Square	F Value	*p*-Value
1	Time of flowering	February	April	September	39	3.96	<0.001 ***
2	Length of leaf (cm)	3.56 ± 0.25	4.36 ± 0.41	4.55 ± 0.31	0.8203	7.364	0.0243 *
3	Width of leaf (cm)	1.1 ± 0.2	0.8 ± 0.1	1 ± 0.1	0.07	3.5	0.0983
3	Length of inflorescence (cm)	4.4 ± 0.7	11.5 ± 1.2	5.45 ± 1.2	44.02	37.45	<0.001 ***
4	Number of flowers per inflorescence	12 ± 4.04	30 ± 7.76	13 ± 4.16	356.8	11.39	<0.001 ***
5	Diameter of flower (mm)	7.2 ± 0.15	7 ± 0.2	8.7 ± 0.25	2.6133	61.9	<0.001 ***
6	Percentage pollen viability	93 ± 3.2	87 ± 3.7	86 ± 4	38.11	28.58	<0.001 ***
7	Pollen output per flower	8040 ± 372.6	6189 ± 454.5	8148 ± 552.9	2583222	11.86	0.00824 **
8	Length and width of the bract (mm)	3.2 ± 0.25 × 2.3 ± 0.19	2.9 ± 0.21 × 2 ± 0.14	3.5 ± 0.27 × 1.9 ± 0.11	0.3733	8.842	0.0163 *
9	Length and width of the porophylls (mm)	2.5 ± 0.05 × 1.55 ± 0.11	1.9 ± 0.09 × 1.4 ± 0.13	2.2 ± 0.17 × 1.4 ± 0.09	0.05444	4.9	0.0548
10	Size of sepal’s outer whorl (mm)	3.6 ± 0.51 × 3.2 ± 0.42	3.6 ± 0.81 × 3.3 ± 0.56	3.3 ± 0.31 × 2.9 ± 0.36	0.05778	4	0.0787
11	Size of sepal’s inner whorl (mm)	5.8 ± 0.42 × 4.6 ± 0.51	6.2 ± 0.67 × 5.5 ± 0.62	5.6 ± 0.32 × 4.9 ± 0.55	0.00778	0.875	0.464
12	Size of petal (mm)	5.75 ± 0.45 × 4.25 ± 0.41	6.25 ± 0.32 × 5.12 ± 0.56	6.15 ± 0.37 × 4.75 ± 0.31	0.09	5.4	0.0456 *
13	Length of pistil (mm)	4.55 ± 0.49	5.17 ± 0.47	4.95 ± 0.37	0.3333	13.04	0.00654 **
14	Length of stamen (mm)	3.55 ± 0.49	3.67 ± 0.41	3.59 ± 0.39	0.2923	2.642	0.15
15	Width of flower (mm)	7.3 ± 0.61	7.9 ± 0.47	7.7 ± 0.31	0.5983	5.543	0.0433 *

*p* is the level of significance (* ≤0.05; ** ≤0.01; *** ≤0.001; without * = non-significant).

**Table 2:** Pattern of flower anthesis and anther dehiscence in *Berberis lycium.*

Variant	Anthesis	Time	Duration	Maximum	Completion	Anther Dehiscence
I	February	08:00 am ± 17 min	2.5 h ± 22 min	76% ± 7.8 at 09:00–16:08 h	Throughout day	During and after dehiscence
II	April	07:00 am ± 24 min	3 h ± 17 min	82% ± 11.7 at 09:30–15:15 h	Throughout day	During and after dehiscence
III	September	06:17 am ± 13 min	3.15 h ± 15 min	79% ± 9.5 at 09:15–15:39 h	Throughout day	During and after dehiscence

**Table 3:** Pollen viability and pollen output of *Berberis lycium.*

S.No	Sample Size (n)	Pollen Viability	Average Value			ANOVA		
			Variant I	Variant II	Variant III	Mean Square	F Value	*p*-Value
1	15	Acetocarmine (%)	91.60 ± 2.10	86.31 ± 7.26	82.52 ± 4.61	321.02	57.61	<0.001
2	15	FDA (%)	90.42 ± 1.94	87.19 ± 5.98	85.73 ± 4.46	86.2	16.09	<0.001
3	20	Pollen output per flower	7979.1 ± 1908	6012 ± 1469.04	8604 ± 1193.88	3.544	52.4	<0.001

**Table 4:** Insect visitors for different morphotypes of *Berberis lycium* and their foraging activity.

S. No.	Insect Visitor	Order	Family	Variant I	Variant II	Variant III	Foraging Activity
1	*Allograpta* sp.	Hymenoptera	Apidae	+	-	+	Nectar
2	*Altica* sp.	Coleoptera	Chrysomelidae	+	-	-	Nectar and pollen
3	*Andrena flavipes*	Hymenoptera	Adrenidae	-	+	-	Nectar
4	*Aphidoidea* (aphids)	Hemiptera	Aphididae	+	-	+	Pollen
5	*Apis cerana*	Hymenoptera	Apidae	+	+	+	Nectar
6	*Apis mellifera*	Hymenoptera	Apidae	+	+	+	Nectar
7	*Bombus trifasciatus*	Hymenoptera	Apidae	+	-	+	Nectar and pollen
8	*Calliphora vomitoria*	Diptera	Calliphoridae	+	-	-	Nectar and pollen
9	*Camponotus pennsylvanicus*	Hymenoptera	Formicidae	+	-	+	Nectar and pollen
10	*Coelixys* sp.	Hymenoptera	Megachilidae	+	+	+	Nectar and pollen
11	*Dodona durga*	Lepidoptera	Riodinidae	+	+	+	Nectar
12	*Eupeodes luniger*	Diptera	Syrphidae	+	-	+	Nectar
13	*Formica fusca*	Hymenoptera	Formicidae	+	+	+	Pollen
14	*Heliophorus sena*	Lepidoptera	Lycaenidae	+	-	+	Nectar
15	*Musa domestica*	Dipteria	Muscidae	+	+	+	Nectar
16	*Plecia* sp.	Dipteria	Bibionidae	+	-	-	Pollen
17	*Praezygaena caschmirensis*	Lepidoptera	Zygaenidae	-	+	-	Pollen
18	*Sarcopha* sp.	Diptera	Sarcophagidae	+	-	-	Pollen

**Table 5:** Comparison for different pollination indices of different insect visitors of *Berberis lycium* (measured following the methodology of Yaqoob and Nawchoo [19]).

Pollinator	Foraging Behavior	Insect Visiting Efficiency	Foraging Speed	Index of Visitation Rate
*Allograpta* sp.	4.1 ± 0.84	0.119 ± 0.09	20.8 ± 5.5	112 ± 23.3
*Apis cerana*	6.3 ± 1.4	0.176 ± 0.11	31 ± 8.2	214 ± 27.7
*Apis mellifera*	5.91 ± 0.82	0.127 ± 0.08	27 ± 8.5	227 ± 31.1
*Bombus trifasciatus*	5.17 ± 2.2	0.227 ± 0.09	19 ± 6.6	204 ± 22.72
*Calliphora vomitoria*	9.17 ± 2.19	0.361 ± 0.163	59.8 ± 16.6	389 ± 49.9
*Coelixys* sp.	1.59 ± 0.12	0.05 ± 0.01	6.98 ± 2.21	36.06 ± 7.72
*Dodona durga*	1.17 ± 0.39	0.09 ± 0.068	3.03 ± 0.97	7.71 ± 2.2
*Eupeodes luniger*	3.61 ± 1.19	0.143 ± 0.11	11.03 ± 2.19	47.07 ± 12.02
*Formica fusca*	1.23 ± 0.29	0.03 ± 0.009	5.72 ± 1.9	10.17 ± 3.3
*Heliophorus sena*	1.03 ± 0.29	0.07 ± 0.052	2.71 ± 0.73	5.51 ± 1.9
*Musa domestica*	8.17 ± 0.87	0.221 ± 0.12	49 ± 11.9	271 ± 33.9
*Praezygaena caschmirensis*	0.55 ± 0.21	0.073 ± 0.021	4 ± 1.7	6.6 ± 2.54
*Sarcopha* sp.	8.09 ± 1.95	0.276 ± 0.128	51.1 ± 13.3	313 ± 36.6

**Table 6:** Reproductive output of *Berberis lycium* under different pollination treatments.

S. No.	Pollination Treatments	Number of Flowers	Number of Fruits Formed			Percentage Fruit Set		
			Variant I	Variant II	Variant III	Variant I	Variant II	Variant III
1	Emasculated and bagged (apomixis)	100	0	0	0	0	0	0
2	Unassisted selfing	100	0	0	0	0	0	0
3	Manual autogamy	100	56	58	59	56	58	59
4	Manual geitonogamy	100	57	61	60	57	61	60
5	Manual cross pollination	100	65	71	68	65	71	68
6	Open pollination	100	31	52	33	31	52	33

**Table 7:** Results of the two-way analysis of variance test (ANOVA) for fruit and seed development across the different variants of *Berberis lycium.*

Parameter		df	Sum Sq	Mean Sq.	F Value	*p*-Value
Fruit	Variants	1	6	6	0.311	0.731
	Treatments	5	11561	2917	105.28	<0.001
Fruit set	Variant	1	4	4	0.273	0.578
	Treatments	5	6081	1122	125.4	<0.001
Seed	Variants	1	2	2.4	0.135	0.744
	Treatments	5	9370	2117	209.2	<0.001
Seed set	Variants	1	2	2.2	0.131	0.593
	Treatments	5	8861	1671	78.23	<0.001

## Data Availability

Not applicable.
