# Correction: Verma et al. Reproductive Biology and Pollination Ecology of *Berberis lycium* Royle: A Highly Valued Shrub of Immense Medicinal Significance. *Plants* 2021, *10*, 1907

**DOI:** 10.3390/plants15132051

**Published:** 2026-07-02

**Authors:** Susheel Verma, Ishfaq Ahmad Wani, Sajid Khan, Supriya Sharma, Priyanka Kumari, Prashant Kaushik, Hamed A. El-Serehy

**Affiliations:** 1 Conservation and Molecular Biology Laboratory, Department of Botany, Baba Ghulam Shah Badshah University, Rajouri 185234, India; waniishfaq680@gmail.com (I.A.W.); sajidkhan717@gmail.com (S.K.); sup.sharma111@gmail.com (S.S.); pk838511@gmail.com (P.K.); 2 Independent Researcher, 46022 Valencia, Spain; prakau@doctor.upv.es; 3 Department of Zoology, College of Science, King Saud University, Riyadh 11451, Saudi Arabia; helserehy@ksu.edu.sa

In the publication [1], there was an error regarding the affiliation for Prashant Kaushik. The updated affiliation should read as follows: Independent Researcher, 46022 Valencia, Spain.

The authors state that the scientific conclusions are unaffected. This correction was approved by the Academic Editor. The original publication has also been updated.
